# The METTL3 RNA Methyltransferase Regulates Transcriptional Networks in Prostate Cancer

**DOI:** 10.3390/cancers14205148

**Published:** 2022-10-20

**Authors:** Daisy B. Haigh, Corinne L. Woodcock, Jennifer Lothion-Roy, Anna E. Harris, Veronika M. Metzler, Jenny L. Persson, Brian D. Robinson, Francesca Khani, Mansour Alsaleem, Atara Ntekim, Srinivasan Madhusudan, Melissa B. Davis, Kristian B. Laursen, Lorraine J. Gudas, Catrin S. Rutland, Michael S. Toss, Nathan Archer, Zsuzsanna Bodi, Emad A. Rakha, Rupert G. Fray, Jennie N. Jeyapalan, Nigel P. Mongan

**Affiliations:** 1 Biodiscovery Institute, University of Nottingham, University Park, Nottingham NG7 2RD, UK; 2 School of Veterinary Medicine and Sciences, University of Nottingham, Sutton Bonington LE12 5RD, UK; 3 Department of Molecular Biology, Umeå University, 901 87 Umeå, Sweden; 4 Department of Biomedical Sciences, Malmö Universitet, 202 04 Malmö, Sweden; 5 Department of Pathology, Weill Cornell Medicine, New York, NY 10065, USA; 6 School of Medicine, University of Nottingham, Nottingham NG7 2RD, UK; 7 Department of Applied Medical Science, Applied College, Qassim University, Unayzah 51911, Qassim, Saudi Arabia; 8 Department of Radiation Oncology, University College Hospital, University of Ibadan, Ibadan 200132, Nigeria; 9 Department of Surgery, Weill Cornell Medicine, New York, NY 10065, USA; 10 Department of Pharmacology, Weill Cornell Medicine, New York, NY 10065, USA; 11 School of Biosciences, University of Nottingham, Sutton Bonington LE12 5RD, UK

**Keywords:** prostate cancer, METTL3, m6A, RNA methylation, therapeutic, molecular signalling, androgen receptor, endocrine driven cancer

## Abstract

**Simple Summary:**

Prostate cancer is driven by androgen receptor-regulated transcription and is a leading cause of cancer deaths. For this reason, androgen deprivation therapies are commonly used to treat advanced prostate cancer. These treatments are often effective for short durations before the emergence of treatment resistance and disease progression to castrate resistant prostate cancer or neuroendocrine-like disease. The aim of this study was to address whether new therapies targeting the epitranscriptome may suppress androgen signalling and thus represent a novel approach to prostate cancer treatment.

**Abstract:**

Prostate cancer (PCa) is a leading cause of cancer-related deaths and is driven by aberrant androgen receptor (AR) signalling. For this reason, androgen deprivation therapies (ADTs) that suppress androgen-induced PCa progression either by preventing androgen biosynthesis or via AR signalling inhibition (ARSi) are common treatments. The *N*6-methyladenosine (m6A) RNA modification is involved in regulating mRNA expression, translation, and alternative splicing, and through these mechanisms has been implicated in cancer development and progression. RNA-m6A is dynamically regulated by the METTL3 RNA methyltransferase complex and the FTO and ALKBH5 demethylases. While there is evidence supporting a role for aberrant METTL3 in many cancer types, including localised PCa, the wider contribution of METTL3, and by inference m6A, in androgen signalling in PCa remains poorly understood. Therefore, the aim of this study was to investigate the expression of METTL3 in PCa patients and study the clinical and functional relevance of METTL3 in PCa. It was found that METTL3 is aberrantly expressed in PCa patient samples and that siRNA-mediated METTL3 knockdown or METTL3-pharmacological inhibition significantly alters the basal and androgen-regulated transcriptome in PCa, which supports targeting m6A as a novel approach to modulate androgen signalling in PCa.

## 1. Introduction

Prostate cancer (PCa) is a leading cause of cancer-related deaths [1]. The androgen receptor (AR) plays a pivotal role in PCa pathogenesis and progression. Given this critical role, androgen deprivation therapies/AR signalling inhibitors (ADTs/ARSis) that target AR function (eg bicalutamide, enzalutamide, darolutamide, and apalutamide) or inhibit androgen production (eg abiraterone) are the mainstay of systemic PCa treatments. Whilst these therapies initially successfully suppress PCa growth and disease progression, the emergence of treatment resistance and progression to castrate resistant PCa (CRPC) are common [2]. Therefore, there is an urgent need to better understand the transcriptional mechanisms involved in CRPC and to identify novel therapeutic approaches to prevent, reverse, or delay disease progression.

*N*6-methyladenosine (m6A) is the most abundant internal epitranscriptomic modification in eukaryotic mRNA. RNA-m6A methylation is mediated by a multiprotein methyltransferase complex, with methyltransferase-like 3 (METTL3) harboring the catalytic component [3,4,5,6]. Through phylogenetic studies, methyltransferase-like 14 (METTL14) was found to be a close homologue of METTL3 and forms a heterodimer with METTL3 [6,7,8,9]. The METTL3-METTL14 heterodimer interacts with additional proteins, including Wilms tumour 1-associated protein (WTAP), RNA-binding motif protein 15 (RBM15), RBM15B, vir-Like m6A methyltransferase associated (VIRMA, also known as KIAA1429), and Zinc Finger CCCH-Type Containing 13 (ZC3H13), all of which are required for m6A methylation [6,8,10,11,12,13]. Moreover, other proteins including the E3 ubiquitin-protein ligase Hakai (HAKAI) and associated proteins, are required for the full methylation ability in *Arabidopsis* [14,15]. Studies also suggest that the mammalian HAKAI homologue, cbl protooncogene like 1 (CBLL1), is also involved in the mammalian m6A methyltransferase complex [16]. The RNA-m6A modification is interpreted by several ‘reader’ proteins and demethylated by ‘eraser’ proteins [17,18,19,20]. RNA-m6A is known to be involved in the regulation of gene expression, splicing, and translation [21,22,23,24].

The m6A modification and its regulators have been implicated in numerous solid and haematological cancers, including recent studies in PCa. The m6A reader protein YTHDF2 was initially implicated in PCa through targeting *miR-493-3p*, reducing cell proliferation and migration of PCa cells and thus suggesting a role for m6A in prostate carcinogenesis [25]. Since then, several studies have identified a role for METTL3 in PCa. METTL3 was found to be up-regulated in PCa patients and be implicated in many pathways involved in PCa development and progression [26,27,28,29]. A recent study by Cotter et al. [30] highlighted a role for METTL3 in treatment resistance in PCa. Despite these advances, the role of METTL3, and by inference m6A, in androgen signalling and CRPC remains poorly understood.

Therefore, to better understand the role of METTL3 in PCa, its expression and function was investigated in both PCa patients and cell lines. Utilising siRNA-mediated depletion and pharmacological inhibition of METTL3, changes in the basal and androgen-regulated transcriptome, alternative splicing, and cellular phenotype were identified. Collectively, these findings support targeting m6A as a novel approach to suppress pro-oncogenic androgen signalling in PCa.

## 2. Materials and Methods

### 2.1. Bioinformatic Analysis of METTL3 in Clinical Specimens

*METTL3* expression and copy-number alterations (CNA) were investigated in the primary adenocarcinoma The Cancer Genome Atlas (TCGA) Firehose Legacy (*n* = 499) [31], metastatic adenocarcinoma SU2C/PCF Dream Team (*n* = 444) [32], and neuroendocrine PCa (NEPC) Multi-Institute (*n* = 114) [33] datasets. The cBioPortal (Memorial Sloan Kettering Cancer Center, New York, USA, https://www.cbioportal.org/, accessed between 1 June 2020 and 1 September 2020) [34,35] was utilised to analyse CNA and alterations in mRNA expression. For mRNA expression data, cBioPortal compared the relative expression of *METTL3* in tumor samples to the *METTL3* expression distribution in the diploid population of samples giving a Z-score. Additionally, the UCSC Xena browser (UCSC Xena, https://xenabrowser.net, University of California, Santa Cruz, USA, accessed between 1 June 2020 and 1 September 2020) [36] was used to further analyse *METTL3* mRNA expression in the TCGA prostate adenocarcinoma dataset [31] in sample type, primary therapy response, and biochemical recurrence (BCR).

### 2.2. Ethics Statement, Tissue Specimens, and Immunohistochemistry

The project was reviewed and approved by the local ethics committees of the University of Nottingham School of Veterinary Medicine and Science (3483 211102; 1578 151019) and Weill Cornell Medicine IRB (protocol#1008011210). The General Data Protection Regulation (GDPR) was applied, and informed consent obtained. The Helsinki Declaration of Human Rights was strictly observed. The prostate specimen tissue microarray (TMA) cohort comprised of non-malignant (*n* = 152) and primary PCa (*n* = 253) specimens in triplicate 0.6 mm formalin-fixed paraffin-embedded (FFPE) cores (Table S1).

Immunohistochemical (IHC) staining of the TMA was performed using the semi-automatic IHC diagnostic system (Ventana Inc., Oro Valley, USA), as previously described [37]. The TMA blocks were cut at 5 µm sections and stained with a METTL3 primary antibody (ab195352 PUR, Abcam, Cambridge, UK, 1:500). High resolution scans of the stained slides were prepared and the nuclear staining in non-malignant or tumour tissue was assessed independently by two pathologists (BDR, FK) using the H-Score system (0–300 range: 1 × (% cells with staining intensity 1) + 2 × (% cells with staining intensity 2) + 3 × (% cells with staining intensity 3)) [38]. H-scores were then trichotomised into low (<200), medium (200–290), and high (>290) METTL3 nuclear expression and correlated with clinical parameters.

### 2.3. Cell Lines and Culture Conditions

The PCa cell lines LNCaP, LNCaP:C4-2, and 22Rv1 were used in this study. Cell line identity was confirmed by genotyping or STR profiling. All cells were cultured at 37 °C and 5% CO_2_ in phenol-red containing RPMI-1640 medium supplemented with 100 U/mL penicillin, 100 µg/mL streptomycin, 1 mM sodium pyruvate, and 10% foetal bovine serum (FBS). For androgen treatment, cells were plated in phenol-red-free RPMI-1640 medium supplemented with 2 mM L-glutamine, 100 U/mL penicillin, 100 µg/mL streptomycin, 1 mM sodium pyruvate, and 10% charcoal-stripped FBS. Synthetic androgen (R1881) in 100% ethanol was added to the well at a final concentration of 1nM, or 100% ethanol was added for vehicle controls. All reagents were purchased from Gibco (Waltham, USA) or Sigma-Aldrich (Saint Louis, USA).

### 2.4. siRNA Mediated Functional Depletion and Pharmaco-Inhibition of METTL3

Functional depletion of *METTL3* in LNCaP:C4-2 and 22Rv1 PCa cell lines was performed using the ON-TARGETplus siRNA SMART pool targeting *METTL3* (#L-005170-02-0005, Horizon Discovery, Cambridge, UK) utilising the DharmaFECT 2 transfection reagent (Horizon Discovery, Cambridge, UK). The ON-TARGETplus non-targeting siRNA control (#D001810-10-05, Horizon Discovery, Cambridge, UK) was used as a negative control, and transfection performed following the manufacturer’s instructions. Cells were transfected with siRNA and incubated for 48 h without R1881 treatment. After 48 h, cells were transfected again with siRNA and treated with 1nM R1881 (Sigma-Aldrich, Saint Louis, USA) or 100% ethanol (vehicle) and incubated for a further 72 h before harvesting for protein and RNA analysis. Functional inhibition of METTL3 in LNCaP:C4-2 and 22Rv1 was conducted using the recently published METTL3 inhibitor STM2457 [39]. The cells were treated with 10 μM STM2457 (Insight Biotechnology, Wembley, UK) or DMSO (vehicle) for 48 h before harvesting for RNA or protein.

### 2.5. Gene Expression Analysis and Western Blotting

For mRNA and protein expression analysis, PCa cells were treated as described above. RNA was isolated using GenElute Mammalian Total RNA Miniprep Kit with on-column DNase treatment (Sigma-Aldrich, Saint Louis, USA). Extracted RNA was subjected to cDNA synthesis using the qScript cDNA Synthesis Kit (Quanta Biosciences, Beverly, USA). Analysis of mRNA expression was conducted by real-time quantitative polymerase chain reaction (RT-qPCR) with LightCycler 480 Probes Master (Roche Diagnostics, Rotkreuz, Switzerland) and hydrolysis probes carried out in a LightCycler 480 II instrument (Roche, Rotkreuz, Switzerland). Taqman probes used in this study were *GAPDH* (Hs03929097_g1), β*-actin* (Hs01060665_g1), *KLK3* (Hs02576345_m1), and *METTL3* (Hs00219820_m1) all purchased from Thermo Fisher Scientific (Waltham, USA). Relative mRNA expression was analysed using the Pfaffl method [40] with either *GAPDH* or β*-actin* used as the housekeeping gene.

For total protein analysis by Western blotting, cells were harvested in SDS final sample buffer (100 mM Tris-HCl pH 6.8, 4% SDS, 20% glycerol) and stored at −80 °C. To isolate nuclear and cytoplasmic protein fractions from cells, NER-PER Nuclear and Cytoplasmic Extraction Reagents (Thermo Fisher Scientific, Waltham, USA) were used and separation performed using the manufacturer’s instructions. Protein samples were diluted with 5X Laemmli loading buffer and boiled at 95 °C for 5 min before loading on an SDS-PAGE gel for protein separation. After this, the proteins were transferred from the gel onto a polyvinylidene difluoride (PVDF) membrane (0.45 µm, Merck, Rahway, USA) via semi-dry blotting. The membrane was blocked using either 5% bovine serum albumin (BSA) or 5% milk and probed overnight with primary antibody at 4 °C. The following antibodies were used in this study: anti-METTL3 (ab195352 PUR; Abcam, Cambridge, UK 5% milk; 1:10,000), anti-AR (sc-816; Santa Cruz, Dallas, USA 5% milk; 1:5000), anti-GAPDH (mAb9484; Abcam, Cambridge, UK, 5% BSA 1:10,000), anti-β-actin (sc-130657; Santa Cruz, Dallas, USA, 1:50,000 or MA5-15739; Invitrogen, Waltham, USA, 1:10,000), and anti-HDAC1 (5356; Cell Signalling Technology, Danvers, USA, 1:1000). Goat anti-mouse (ab97023; Abcam, Cambridge, UK, 1:10,000–50,000 or Sc-2005; Santa Cruz, Dallas, USA, 1:10,000–50,000) and Goat anti-rabbit (ab6721; Abcam, Cambridge, UK, 1:10,000-50,000 or Sc-2004; Santa Cruz, Dallas, USA, 1:10,000–50,000) secondary antibodies were used for 1 h at room temperature, the signal detected using Amersham ECL Prime reagent (GE Healthcare, Chicago, USA) and imaged using a ChemiDoc MP Imaging System (Bio-Rad, Hercules, USA). Full, annotated Western blot images are available in Figures S1–S9.

### 2.6. RNA-seq and Splicing Analysis of siRNA Depletion or Pharmaco-Inhibition of METTL3

RNA-seq analysis (Novogene, Cambridge, UK) was performed on LNCaP:C4-2 and 22Rv1 PCa cells treated with siSCR control, siMETTL3 in vehicle (ethanol; basal) or androgen (1nM R1881) conditions. Additionally, RNA-seq was performed on LNCaP:C4-2 and 22Rv1 treated with vehicle (DMSO) or 10 μM STM2457. The obtained Fastq files were quality processed (phred score >30 retained) and adapters trimmed using the Trim Galore wrapper (GitHub, Inc., San Francisco, USA, https://github.com/FelixKrueger/TrimGalore, accessed between 1 November 2021 and 1 January 2022) for FastQC and Cutadapt. The QC-processed reads were aligned to the human Ensembl annotated reference genome (GRCh38) using the STAR aligner (GitHub, Inc., San Francisco, USA) [41]. The differential gene expression between conditions was quantified using FeatureCounts (The Walter and Eliza Hall Institute of Medical Research, The University of Melbourne, Parkville, Australia) [42] and DESeq2 (Huber Lab, European Molecular Biology Laboratory, Heidelberg, Germany) [43]. Genes were then filtered by fold change (FC) ±1.5 and false discovery rate (FDR) < 0.05. RNA-seq data was also analysed to assess differential alternative splicing using replicate multivariate analysis of transcript splicing (rMATS, version 3.2.5, Xing Lab, Children’s Hospital of Philidephia, Philadelphia, USA) [44]. Significant differential alternative splicing was sorted based on the difference in percentage spliced in (dPSI) ≥ 5% and FDR < 0.05. KEGG pathway analysis was conducted using gene symbol of the significant differentially expressed genes (DEGs) and differentially spliced genes (DSGs) utilising the Web-Based Gene Set AnaLysis Toolkit (WebGestalt, http://www.webgestalt.org/, Zhang Lab, Baylor College of Medicine, Houston, USA, accessed between 1 March 2022 and 1 October 2022) [45]. Unsupervised hierarchical clustering analysis of normalised counts of AR-regulated genes [46] was completed using Cluster 3.0 (Lawrence Berkeley National Laboratory, Berkeley, USA) [47] and heatmaps generated using Java Treeview (Free Software Foundation Inc., Boston, USA). METTL3-regulated androgen responsive genes were identified using Venny (https://bioinfogp.cnb.csic.es/tools/venny/, accessed between 1 March 2022 and 1 October 2022) by finding unique or overlapping differentially expressed genes in the siSCR Veh vs. R1881 and siMETTL3 Veh vs. R1881 conditions.

### 2.7. Evaluation of Phenotypic Effects

To determine the effect of METTL3 inhibition on proliferation, LNCaP:C4-2 and 22Rv1 PCa cells were plated in phenol-red free RPMI with 10% charcoal-stripped FBS and treated with vehicle (100% DMSO) or 10 μM STM2457 for 3 and 6 days. DNA content was subsequently quantified using the CyQUANT Direct Cell Proliferation Assay (Invitrogen, Waltham, USA) performed following the manufacturer’s instructions, wavelength read (Varioskan Flash plate reader; Thermo Fisher Scientific, Waltham, USA), and the relative DNA content calculated.

To assess in vitro invasion, cell culture inserts (Corning, New York, USA) were coated with Matrigel (Corning, New York, USA) diluted in coating buffer and allowed to set for 24 h at 37 °C. LNCaP:C4-2 and 22RV1 cells were seeded on top of the Matrigel coating in FBS-free, phenol-red-free RPMI-1640 medium. In the well below the insert, phenol-red-free RPMI-1640 medium containing 10% FBS was added to the wells. The cells were then treated with vehicle (100% DMSO) or 10 μM STM2457 and left to incubate for 24 h at 37 °C before fixing and staining invaded cells using methanol and 0.4% crystal violet. Stained cells were imaged using an inverted microscope (Leica, Wetzlar, Germany), the cell number was manually assessed and relative invasion, compared to control treated cell lines, was calculated.

### 2.8. Statistical Analysis

Statistical analysis was carried out using GraphPad Prism (Graphpad Software Inc., San Diego, USA), SPSS v26.0 (IBM, Armonk, USA) statistical software, or VassarStats Website for Statistical Computation (http://vassarstats.net/, accessed between 1 June 2021 nd 1 September 2021). For comparison of two means, *t*-tests were carried out. For comparison of multiple means, a one-way analysis of variance (ANOVA) was performed. The chi-square test (χ2) was performed to analyse relationships between expression and categorical variables. The *p*-values ≤ 0.05 were considered statistically significant.

## 3. Results

### 3.1. Expression of METTL3 in PCa Patients and Cell Lines

**Figure 1:**
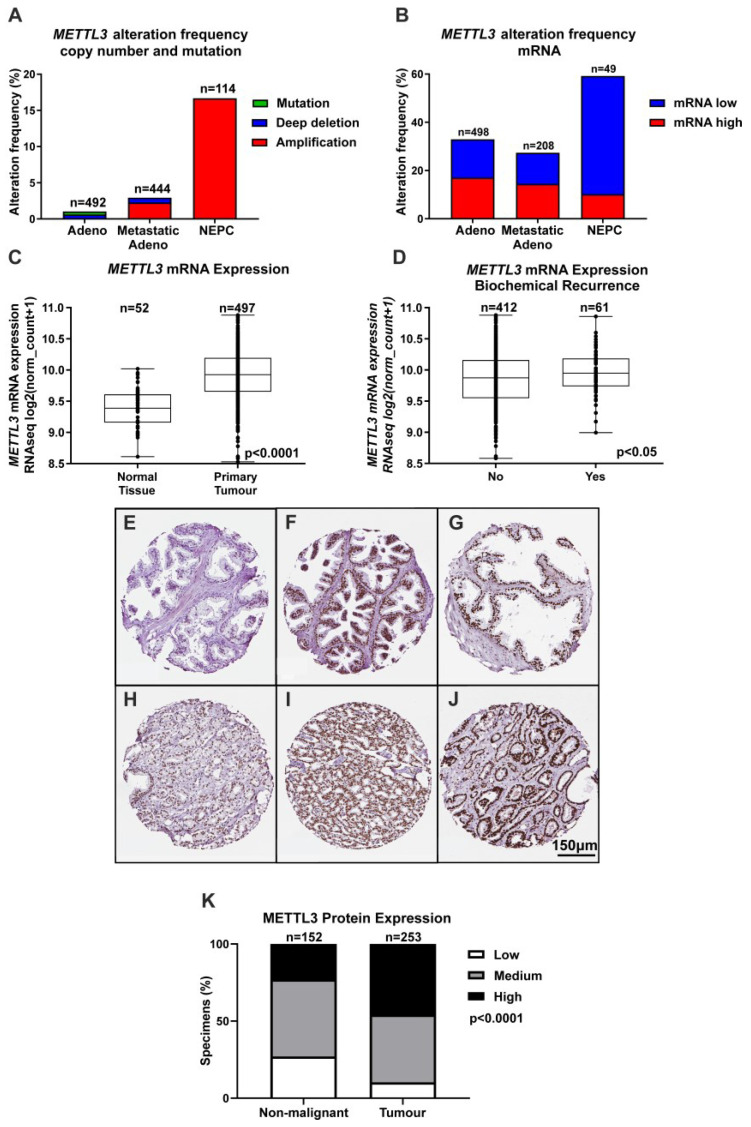
*METTL3* expression in PCa patients. Bioinformatic analysis using multiple PCa datasets from the cBioPortal revealed *METTL3* is altered in PCa (z score threshold ±1) (**A**,**B**). *METTL3* expression was investigated further in normal and primary tumour specimens (**C**) and between patients without or with BCR (**D**) in the TCGA cohort using UCSC Xena. IHC analysis in prostate specimens identified a range of METTL3 protein expression in non-malignant (**E**–**G**) and tumour (**H**–**J**) specimens. A comparison of non-malignant and tumour METTL3 expression identified higher expression in tumour specimens (**K**). Adeno = adenocarcinoma, NEPC = neuroendocrine PCa.

Given the important role for m6A in transcription and translation and evidence implicating METTL3 in many cancer types, bioinformatic analyses of publicly available clinical genomic datasets was undertaken to understand the role of METTL3 in PCa. While CNA were rare in primary prostate adenocarcinoma samples (~1% of cases), *METTL3* CNA were more common in metastatic adenocarcinoma samples (~3% of cases), and NEPC (~17% of cases) (Figure 1A). Analysis of *METTL3* mRNA expression alterations revealed that ~32% of adenocarcinoma samples possessed altered *METTL3* mRNA expression, with 79/498 cases (~15%) having low *METTL3* expression, and 85/498 cases (~17%) possessing elevated *METTL3*. For metastatic adenocarcinoma, 27/208 cases (~13%) had low *METTL3* expression and 30/208 cases (~14%) had elevated *METTL3* expression. Strikingly, the NEPC dataset also possessed the highest *METTL3* mRNA alteration frequency at ~59%, with low *METTL3* mRNA expression in 24/49 cases (~49%) and high *METTL3* mRNA expression in 5/49 cases (~10%) (Figure 1B). *METTL3* expression was significantly higher in primary tumour compared to non-malignant prostate tissue (Figure 1C; *p* < 0.0001), and significantly higher in cases with BCR (Figure 1D; *p* < 0.05). *METTL3* was lower in cases with complete or partial remission/response to primary therapy in comparison to cases with stable disease (Figure S10; *p* < 0.01).

To further investigate the expression and clinical relevance of METTL3 in PCa patients, IHC analysis of METTL3 expression in non-malignant and PCa patient samples was performed. A range of staining was observed in the nuclear compartment of prostate glandular cells (Figure 1E–J). Consistent with mRNA expression, METTL3 protein expression was also significantly higher in tumour as compared with non-malignant specimens (Figure 1K and Table S2; *p* < 0.0001).

**Figure 2:**
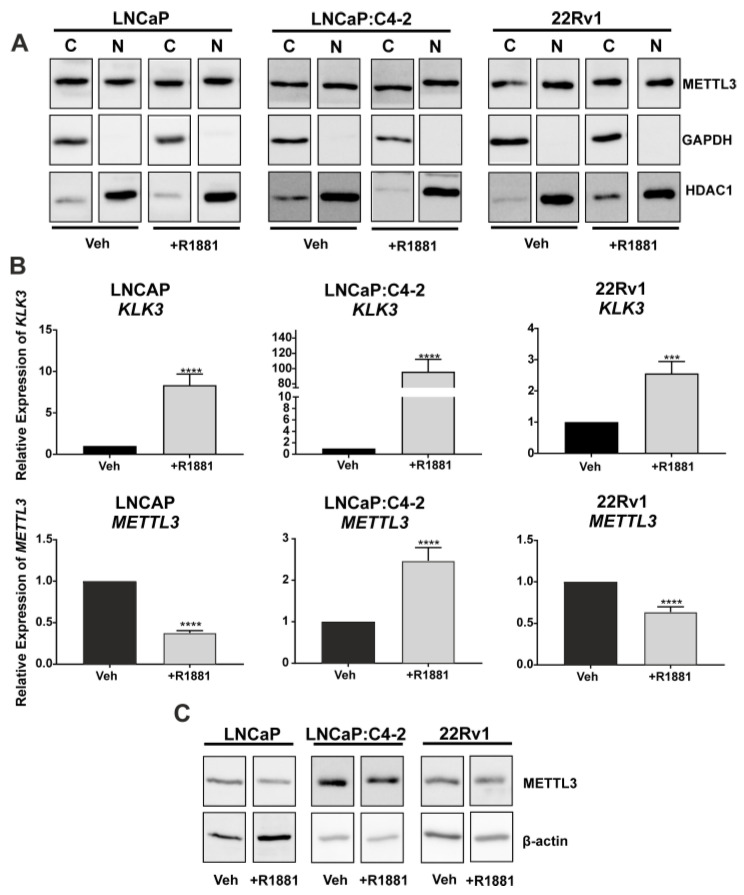
METTL3 expression in PCa cell lines. (**A**) METTL3 protein expression was examined in the nuclear and cytoplasmic compartments of PCa cell lines LNCaP, LNCaP:C4-2, and 22Rv1. Cytoplasmic and nuclear loading controls used were GAPDH and HDAC1, respectively. Androgen regulation of *KLK3* and *METTL3* mRNA (**B**) and METTL3 protein (**C**) expression was analysed. Veh = vehicle, C = cytoplasmic, N = nuclear. *** *p* ≤ 0.001, **** *p* ≤ 0.0001. Molecular weights for proteins are indicated in the full annotated Western blot images (Figures S1–S6).

To assess the translational relevance of PCa cell line models, METTL3 protein expression and sub-cellular localisation were investigated. METTL3 was expressed in both the cytoplasmic and nuclear protein fractions, and the distribution of METTL3 protein between compartments was not altered by androgen (R1881) treatment (Figure 2A). The effect of androgen on METTL3 protein and mRNA expression was next assessed in PCa cell lines. The induction of *KLK3* in the PCa cell lines was confirmed (Figure 2B; *p* < 0.05). *METTL3* mRNA expression was significantly up-regulated by androgen in LNCaP:C4-2, but significantly reduced by androgen in LNCaP and 22Rv1 (Figure 2B; *p* < 0.05). At the protein level, treatment with R1881 in LNCaP led to a reduction of METTL3 protein, but expression remained unchanged in LNCaP:C4-2 and 22Rv1 (Figure 2C). This indicates androgen regulation of *METTL3* expression in PCa cells is cell line and context dependent.

### 3.2. Functional Inhibition of METTL3 Alters PCa Cell Line Transcriptome and Splicing

**Figure 3:**
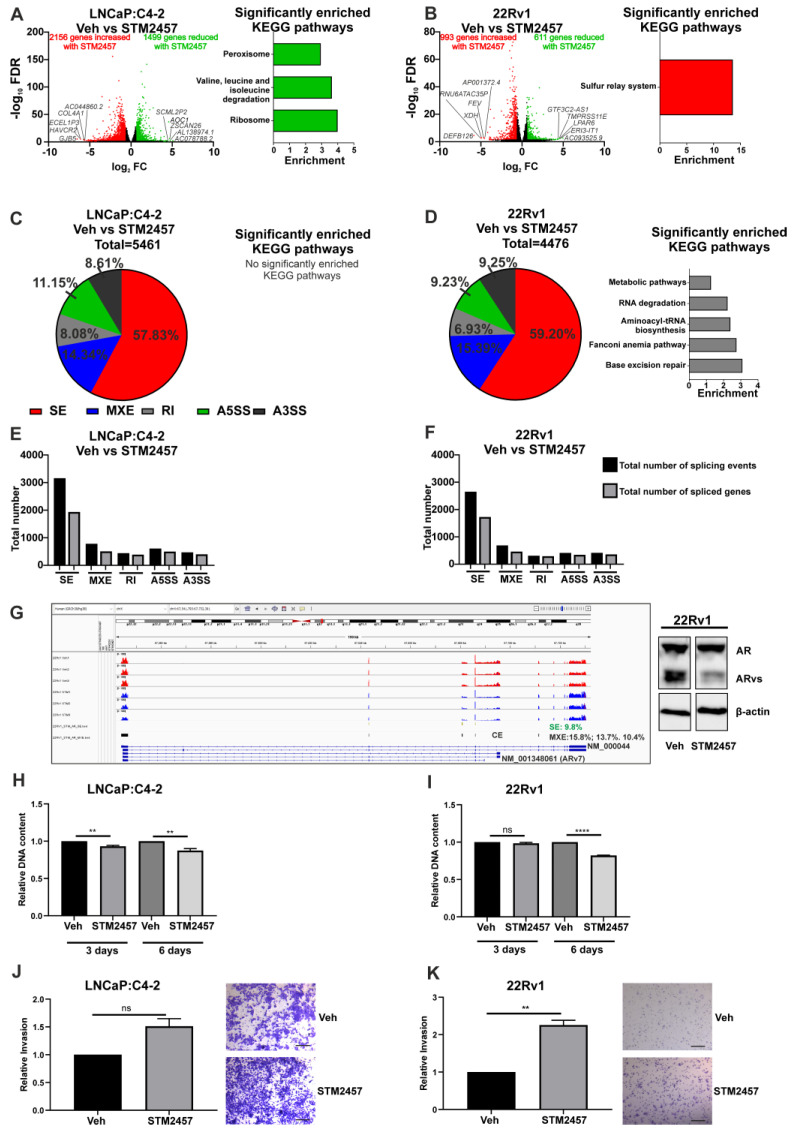
Pharmacological inhibition of METTL3 by STM2457 regulates transcriptional and alternative splicing programmes and cellular phenotype in PCa cell lines. PCa cells treated with STM2457 (10 μM) were analysed by RNA-seq and the DEGs and DSGs determined. The METTL3-regulated differential gene expression and significantly enriched KEGG pathways in LNCaP:C4-2 (**A**) and 22Rv1 (**B**) are shown. Genes with significantly higher expression following METTL3 inhibition are red and genes with significantly lower expression following METTL3 inhibition are green. Non-significantly differentially expressed genes are plotted in black. METTL3-regulated differential splicing events in LNCaP:C4-2 (**C**) and 22Rv1 (**D**) are shown. The total number of significant splicing events and genes in LNCaP:C4-2 (**E**) and 22Rv1 (**F**) are shown. Normalised expression coverage for vehicle (red) and STM2457 (blue) treated 22Rv1 cells is presented using the Integrated Genome Viewer (**G**) [48]. The effect of the STM2457 METTL3 inhibitor on *AR* transcript expression was examined using the rMATs tool. Significant (FDR < 0.05) alterations of differential splicing, both mutual exclusive exon (indicated with black bars, 15.8, 13.7% and 10.4% dPSI) and skipped exon splicing events (9.8% dPSI, green bars), including affecting the cryptic exon (CE) associated with *AR-v7* (NM_001348061) was observed. Treatment of 22Rv1 with STM2457 results in a decrease in the protein expression of AR variants (ARvs) (**G**). The effect of METTL3 inhibition on proliferation (**H**,**I**) and in vitro invasion (**J**,**K**) was also assessed. The scale bar represents 200μm. Significant gene expression: FC ± 1.5 and FDR < 0.05. Significant splicing events: dPSI ≥ 5% and FDR < 0.05. Veh = vehicle, FC = fold-change, FDR = false discovery rate. SE = skipped exon, MXE = mutually exclusive exon, RI = retained intron, A5SS = alternative 5′ splice site, A3SS = alternative 3′ splice site. ** *p* ≤ 0.005, **** *p* ≤ 0.0001. Molecular weights for proteins are indicated in the full annotated Western blot images (Figure S7).

To understand the role of METTL3 in PCa, the functional effects of pharmacological inhibition of METTL3 was investigated. Experiments were conducted using the recently reported METTL3 inhibitor STM2457 [39] on the LNCaP:C4-2 and 22Rv1 cell lines. RNA-seq was used to analyse the effect of a 48-h STM2457 treatment on these cell lines (Figure 3A,B). In LNCaP:C4-2, STM2457 altered the expression of 3655 genes, with expression of 1499 genes reduced by STM2457 and 2156 genes up-regulated (Figure 3A and Table S3). In 22Rv1, STM2457 regulated 1604 genes, with expression of 611 genes reduced and 993 increased by STM2457 (Figure 3B and Table S3). Interestingly, 156 genes were commonly down-regulated, and 423 genes were commonly up-regulated by STM2457 in LNCaP:C4-2 and 22Rv1. Despite the large number of DEGs, few KEGG pathways were found to be enriched with up- or down-regulated genes with STM2457 treatment (Figure 3A,B and Table S4).

Given the evidence that m6A plays a role in regulating splicing, the effect of METTL3 inhibition on genome wide splicing in STM2457 treated LNCaP:C4-2 and 22Rv1 cells was investigated. METTL3 inhibition by STM2457 induced 5461 and 4476 significant differential splicing events, affecting 2947 genes and 2591 genes, respectively, in LNCaP:C4-2 (Figure 3C,E and Table S5) and 22Rv1 (Figure 3D,F and Table S6). Whilst all five splicing types (alternative 5′ and 3′ splice site, mutually exclusive exons, skipped exons and retained introns) were identified, the majority of splicing events in both cell lines were skipped exon events (Figure 3C–F). Interestingly, pathway analysis revealed that the identified DSGs in 22Rv1 with METTL3 inhibition were significantly enriched in pathways such as Fanconi anemia pathway, metabolic pathways, base excision repair, aminoacyl-tRNA biosynthesis, and RNA degradation (Figure 3D and Table S6). No pathways were significantly enriched with spliced genes following STM2457 treatment in LNCaP:C4-2 (Figure 3C and Table S5). Notably, splicing of 994 genes (21.9%) was commonly altered in STM2457 treated LNCaP:C4-2 and 22Rv1, suggesting that many of the DSGs are cell line dependant. The effect of the STM2457 on *AR* transcript expression in 22Rv1 was examined (Figure 3G). Significant alterations of differential splicing, both mutual exclusive exon and skipped exon splicing events, including affecting the cryptic exon (CE) associated with *AR-v7* (NM_ 001348061) was observed. In agreement with this, treatment of 22Rv1 with STM2457 resulted in a decrease in the protein expression of AR variants (ARvs), thereby increasing the ratio of AF-FL to ARvs. This shows that inhibition of METTL3 methyltransferase function results in changes in DEGs and DSGs through either direct or indirect regulation by METTL3.

Next, the effect of the STM2457 METTL3 inhibitor on PCa cell proliferation and invasion was investigated. STM2457 reduced proliferation of both LNCaP:C4-2 and 22Rv1 over 6 days (Figure 3H,I). However, whilst STM2457 had no significant effect on LNCaP:C4-2 invasion, METTL3 inhibition by STM2457 enhanced 22Rv1 invasion (Figure 3J,K). Thus, METTL3 inhibition affects both PCa cell proliferation and invasion.

### 3.3. Depletion of METTL3 Alters the Androgen-Regulated PCa Transcriptome and Splicing

**Figure 4:**
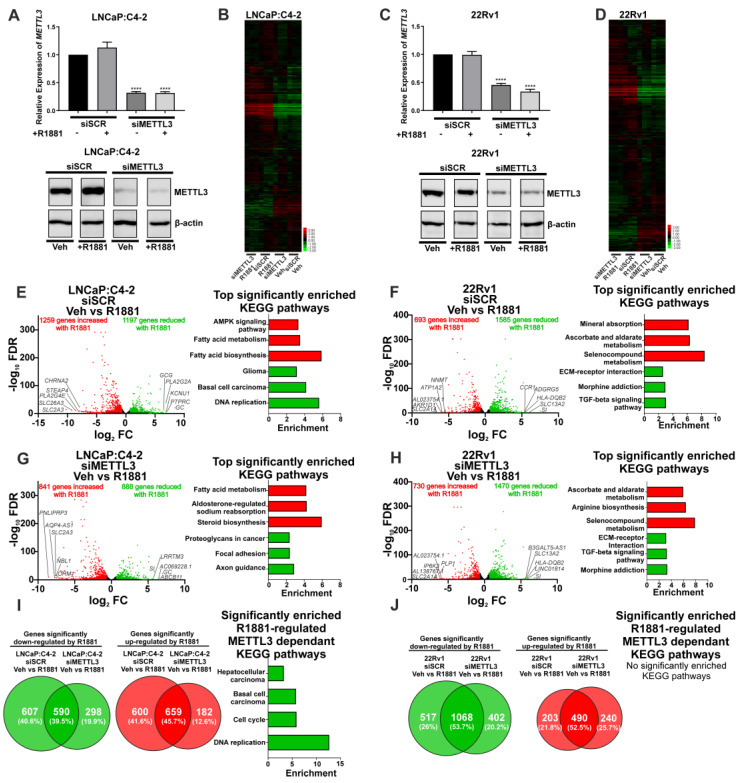
Knockdown of *METTL3* influences androgen-induced transcriptional programmes in PCa cell lines. PCa cells treated with −/+ 1nM R1881 and −/+ si*METTL3* were analysed by RNA-seq and the DEGs determined. *METTL3* knockdown in LNCaP:C4-2 (**A**) and 22Rv1 (**C**) −/+ R1881 was confirmed by qRT-PCR and Western blotting. Heatmap of AR-regulated genes LNCaP:C4-2 (**B**) and 22Rv1 (**D**) siSCR and siMETTL3 treated with Veh or R1881. The androgen-induced differential gene expression and top significantly enriched KEGG pathways in siSCR Veh vs. R1881 and siMETTL3 Veh vs. R1881 in LNCaP:C4-2 (**E**,**G**) and 22Rv1 (**F**,**H**) is shown. Genes with significantly higher expression with R1881 treatment are indicated in red and genes significantly lower with R1881 treatment are indicated in green. Non-significantly DEGs are plotted in black. Common and uniquely regulated genes in siSCR vs. siMETTL3 and enriched KEGG pathways with METTL3 dependant R1881 regulated genes in LNCaP:C4-2 (**I**) and 22Rv1 (**J**) are shown. Significant gene expression: FC ±1.5 and FDR < 0.05. **** *p* ≤ 0.0001. Molecular weights for proteins are indicated in the full uncropped annotated Western blot images (Figures S8 and S9).

Next, siRNA-mediated functional depletion of *METTL3* was conducted to determine its role in the androgen-regulated transcriptome and splicing in PCa cells. Knockdown of *METTL3* using siRNA was conducted to analyse the basal (vehicle) and androgen-regulated transcriptome (R1881) in LNCaP:C4-2 and 22Rv1. Knockdown of *METTL3* at the mRNA and protein levels was confirmed (Figure 4A,C) and samples were analysed by RNA-seq and rMATs to identify DEGs, DSGs, and pathways enriched with regulated genes.

*METTL3* knockdown was associated with a limited number of unique and common DEGs in vehicle treated LNCaP:C4-2 and 22Rv1 cells (Figure S11A,B and Table S7). Given the limited number of identified METTL3-regulated genes, no significantly enriched pathways were identified in 22Rv1 (Figure S11B and Table S8). However, METTL3-regulated genes in the LNCaP:C4-2 basal transcriptome were associated with pathways linked to MAPK signalling, HIF-1 signalling, drug metabolism, and steroid hormone biosynthesis (Figure S11A and Table S8). A larger number of DSGs on the basal transcriptome were identified than DEGs following *METTL3* knockdown, and pathways enriched with spliced genes were associated with metabolism and autophagy (Figure S11C–F, Tables S9 and S10).

**Figure 5:**
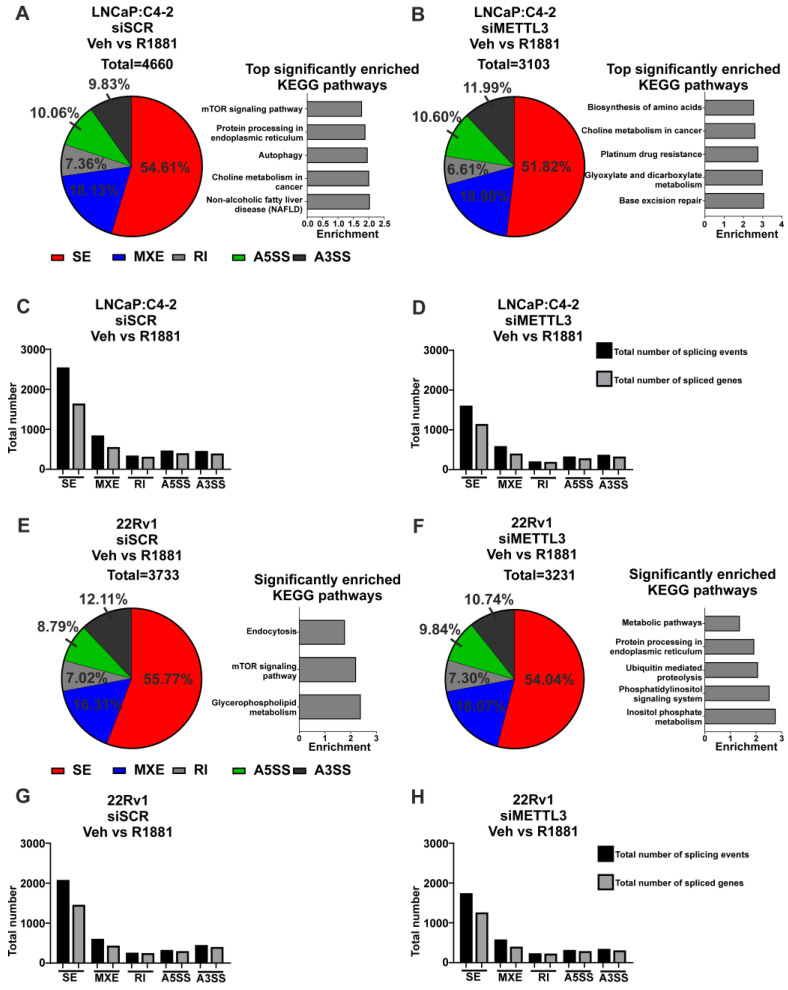
Knockdown of *METTL3* influences androgen-induced splicing in PCa cell lines. PCa cells treated with −/+ 1nM R1881 and −/+ siMETTL3 were analysed by RNA-seq and the DSGs determined. The significant androgen-regulated differential splicing events, genes, and top significantly enriched KEGG pathways in siSCR Veh vs. R1881 and siMETTL3 Veh vs. R1881 in LNCaP:C4-2 (**A**–**D**) and 22Rv1 (**E**–**H**) are shown. Significant splicing events: dPSI ≥ 5% and FDR < 0.05. Veh = vehicle, FC = fold-change, FDR = false discovery rate. SE = skipped exon, MXE = mutually exclusive exon, RI = retained intron, A5SS = alternative 5′ splice site, A3SS = alternative 3′ splice site.

As androgens and AR signalling are critical in PCa initiation and progression, the effect of siRNA-mediated *METTL3* depletion on the androgen-induced transcriptome was examined in LNCaP:C4-2 and 22Rv1 cells. The effect of METTL3 depletion on androgen-regulated gene expression and splicing was determined (Figure 4 and Figure 5). This approach enabled the identification of METTL3-sensitive, androgen-regulated genes in LNCaP:C4-2 and 22Rv1 (Figure 4B,D).

In LNCaP:C4-2, 2456 genes were differentially regulated by androgen (Figure 4E and Table S11). Of these 2456 androgen-regulated genes, the expression of 1207 genes (49.1%) were also dependent upon METTL3 (Figure 4B,E,G,I and Table S11). In 22Rv1, which expresses AR variants such as ARv7, 2278 androgen-regulated genes were identified (Figure 4F and Table S13), of which 720 genes (31.6%) required METTL3 (Figure 4F,H,J and Table S13). Pathway analysis identified that networks including metabolic pathways, focal adhesion, and axon guidance pathways were commonly regulated by androgen in both LNCaP:C4-2 and 22Rv1 (Tables S12 and S14). Interestingly, a number of pathways were only enriched when METTL3 was depleted, whilst others required METTL3 for androgen regulation, suggesting that METTL3 may regulate a subset of androgen regulated transcription (Figure 4I, Tables S12 and S14).

Next, the role of METTL3 in androgen-regulated alternative splicing was investigated. In LNCaP:C4-2, a total of 4660 alternative splicing events (with 2648 genes associated with these events) were induced by androgen, whereas only 3103 events (with 1983 genes associated with these events) were induced by androgen with *METTL3* depletion (Figure 5A–D, Tables S15 and S16). Pathway analysis revealed eight pathways that were significantly enriched with genes alternatively spliced with androgen treatment, whereas 32 pathways were identified as significantly enriched with genes differentially spliced with androgen treatment upon *METTL3* depletion (Figure 5A,B, Tables S15 and S16). Many pathways involved in cancer were identified to be enriched by genes when *METTL3* is depleted, including genes involved in the PCa KEGG pathway, such as *TMPRRS2* and *KLK3*. This suggests that METTL3 regulates the normal splicing and function of cancer-associated genes.

In 22Rv1, 3733 alternative splicing events were induced by androgen (with 2371 genes associated with these events), whereas only 3231 alternative splicing events (2087 genes associated with these events) were induced with androgen in cells with *METTL3* depletion (Figure 5E–H, Tables S17 and S18). Pathway analysis revealed three pathways that were significantly enriched with genes alternatively spliced with androgen treatment, whereas five pathways were significantly enriched with genes differentially spliced with androgen treatment upon *METTL3* depletion (Figure 5E,F, Tables S17 and S18). There was no overlap in the significantly enriched pathways with or without *METTL3* knockdown.

## 4. Discussion

The METTL3 RNA-m6A methyltransferase has been reported to be involved in the development and progression of many cancer types, including PCa [26,27,28,29,30,49,50,51]. This study aimed to investigate the functional and clinical relevance of METTL3 in PCa patients and cell lines. Additionally, this study addressed the role of METTL3 in the androgen-regulated transcriptome.

Analysis of publicly available PCa datasets confirmed that *METTL3* copy number and mRNA expression is altered frequently in PCa patients, including in advanced tumour types, supporting a role for METTL3 in PCa progression. The heterogeneity of *METTL3* mRNA expression in PCa patients suggests METTL3 has complex roles in PCa and does not function uniquely as an oncogene or tumour suppressor. Crucially, in the context of METTL3 as a potential therapeutic target, in the sub-set of patients with aggressive disease, *METTL3* mRNA expression was elevated in cases with BCR, and lower in cases with complete or partial remission/response in comparison to cases that exhibited stable disease after primary therapy. This suggests a role for METTL3 in therapy resistance. This is supported by recent studies that show both overall survival and progression-free survival to be significantly worse in PCa patients with high METTL3 expression [26,29,50,51,52]. This study’s findings that higher *METTL3* mRNA expression in primary tumour samples compared to normal prostate tissue, is consistent with recently published studies [27,28,49,53]. Collectively these findings suggest that increased METTL3 expression may play a pro-tumourigenic role in PCa.

Investigation of METTL3 protein expression in non-malignant prostate and PCa specimens identified that METTL3 is expressed in the nuclei of prostate cells in patient specimens. Importantly, METTL3 protein expression was significantly higher in tumour specimens as compared to non-malignant prostate specimens, as previously reported in smaller cohorts [26,50,51]. Whilst the IHC analysis of METTL3 expression in patient specimens indicates that METTL3 is predominantly expressed in the nucleus, METTL3 has also been reported to have both nuclear and cytoplasmic functions [54]. However, the sub-cellular localisation of METTL3 in PCa cells was unknown. To address this, the expression and sub-cellular localisation of METTL3 protein expression was assessed. Interestingly, METTL3 protein is expressed in both the cytoplasm and nucleus of the androgen dependent LNCaP and castrate resistant LNCaP:C4-2, and 22Rv1 PCa cell lines. This study is consistent with previous findings that also observed METTL3 expression in the cytoplasm, suggesting the dynamic RNA-m6A modification can be catalysed on nuclear exported RNA. This also supports reports that METTL3 has an alternative role in translational regulation in the cytoplasm [54]. While the cytoplasmic roles of METTL3 warrant further studies, the focus of this study was on nuclear METTL3 function in the basal and androgen-regulated gene expression and splicing given the crucial role of androgen signalling and AR in prostate carcinogenesis, disease progression and treatment response. Therefore, it is notable that METTL3 expression was androgen-regulated in a cell type specific manner.

The recently reported METTL3 inhibitor, STM2457, has been shown to reduce acute myeloid leukaemia cell proliferation [39]. However, the effect of METTL3 inhibition by STM2457 in PCa remained unknown. This study identified that STM2457 altered expression of a larger number of DEGs as compared with siRNA-mediated *METTL3* depletion. This may be attributable to their distinct mechanisms of action, as STM2457 effects immediate pharmacological inhibition of the METTL3-METTL14 complex, whereas siRNA mediated depletion is achieved over a longer time period. Future studies should address the kinetics of dynamic regulation of transcriptome-wide m6A distribution in response to STM2457. Given that the m6A modification and the associated regulatory proteins, such as YTHDC1, are involved in alternative splicing, the effect of METTL3 inhibition by STM2457 on genome wide alternative splicing was investigated in PCa cell lines, which has not previously been reported [21,22,55]. Inhibition of METTL3 resulted in a large number of differential splicing events, with the majority being skipped exon events. Importantly, significant alterations of differential splicing of AR, including affecting the cryptic exon (CE) associated with *AR-v7* was observed. In agreement with this, treatment of 22Rv1 with STM2457 resulted in a decrease in the protein expression of AR variants (ARvs), thereby increasing the ratio of AF-FL to ARvs, suggesting that METTL3 regulates AR splicing in 22Rv1 cell line. This supports a role for METTL3 in the regulation of splicing of the basal transcriptome in the LNCaP:C4-2 and 22Rv1 PCa cell lines. This shows that inhibition of METTL3 methyltransferase function results in changes in DEGs and DSGs through either direct or indirect regulation by METTL3. Despite METTL3 inhibition inducing many differentially expressed and spliced genes, few statistically significant enriched KEGG pathways were identified. This is consistent with estimates that ~60% of transcripts harbour m6A, therefore pharmacological inhibition of the METTL3-METTL14 complex by STM2457 would induce transcriptome-wide reprograming of expression of m6A-regulated transcripts, rather than affecting specific pathways.

Given that androgen signalling is essential for PCa carcinogenesis, progression, and therapeutic response, and persists in CRPC [56], a comparison of the androgen-induced transcriptome following *METTL3* knockdown was completed to identify genes regulated by both m6A and androgen. This identified a number of significant DEGs regulated by androgen, independent of *METTL3* knockdown in both LNCaP:C-42 and 22Rv1. Interestingly, a number of genes were identified to be uniquely differentially expressed under basal levels of METTL3, suggesting that METTL3 may be required for regulating the expression of these genes. Several genes were induced by androgen only in the absence of METTL3. This suggests that either these genes are regulated only by METTL3, or that METTL3 may play a role in impairing the induction of these genes under normal conditions. Further studies are required to address whether METTL3 plays a role in attenuating a subset of androgen regulated genes. In addition to their role in transcriptional regulation, nuclear receptors, including the AR, have been shown to recruit coregulators that influence alternative splicing of target genes [57,58]. This is particularly relevant to PCa where full-length AR and the pathogenic truncated variant AR-v7 regulate distinct splicing networks [59]. Therefore, this study investigated the role of METTL3 on androgen-induced splicing. *METTL3* knockdown resulted in a lower number of androgen-regulated total splicing events, compared with the scramble control. Detailed analysis identified more significantly enriched pathways associated with androgen-regulated differential splicing than DEGs. This supports a role for METTL3 in the regulation of splicing of the basal and androgen-regulated transcriptome. In LNCaP:C4-2 many genes involved in the PCa KEGG pathway, including *KLK3* and *TMPRSS2,* were differentially spliced, further implicating METTL3 in PCa.

While the precise roles of m6A in the regulation of splicing has been controversial [60,61,62], this may reflect differences in biological contexts and experimental models. The complexity of the multifaceted roles of m6A in RNA stability, exon selection and translation is widely accepted [63]. We and others have implicated RNA-m6A methylation as an evolutionary-conserved mechanism involved in the expression and splicing of a subset of key genes in vivo [22,64]. Consistent with this, the accumulation of m6A is implicated in final exon selection [65] and intron/exon inclusion [66]. Furthermore, there is compelling evidence supporting a role for m6A binding proteins in regulation of exonisation and splicing [67,68]. Supporting these findings, this study shows siRNA mediated depletion and pharmacological inhibition of METTL3 had modest effects on differential gene expression, but dramatically alters splicing of many genes. This suggests that targeting METTL3 function may regulate a subset of androgen-regulated transcription and splicing in PCa cells. This is particularly relevant for the treatment of advanced PCa, given the importance of AR [56] and pathogenic AR-variants which cooperate with full-length AR [69] and are key determinants of treatment response [70,71,72].

The effect of STM2457 on PCa cell phenotype was also investigated. STM2457 decreased PCa cell proliferation, which is consistent with the effect of functional depletion of METTL3 identified in previous studies [26,27,29,50,51]. This supports a pro-proliferative role for METTL3 in PCa. However, STM2457 significantly increased in vitro cell invasion in the castrate-resistant and enzalutamide-resistant 22Rv1 cell line which expresses full length AR and AR-variants, but not in the LNCaP:C4-2 castrate-resistant, enzalutamide-sensitive PCa cells which only express full-length AR [73]. This contrasts with previous studies in other PCa cell line models that showed that *METTL3* knockdown reduced invasion [27,50,51]. Given the identification here of pathways enriched with differentially expressed and spliced genes associated with cancer, it is plausible that METTL3 expression is higher in PCa and associated with BCR and therefore such patients may benefit from METTL3 inhibition. Interestingly, differences were identified between the 22Rv1 and LNCaP:C4-2 cell lines. The major clinically relevant differences between these two castrate-resistant PCa cell line models is that 22Rv1, but not LNCaP:C4-2, expresses AR-variants and is enzalutamide resistant. Future preclinical studies should explore whether METTL3 inhibition may be an effective approach to suppress androgen signalling in PCa lacking AR-variant expression. In this way METTL3 inhibition could be therapeutically beneficial for a subset of PCa patients.

## 5. Conclusions

This study identifies a complex but important role for METTL3 in PCa, including in androgen-regulated differential gene expression and splicing. Given the identification of differences in METTL3-regulated differential gene expression and splicing between cell lines, further studies are warranted to explore whether the transcriptome-wide distribution of m6A methylation differs in PCa patients and if this has prognostic value. Such studies would inform on the relevance of therapeutic targeting of METTL3 in preclinical trials for PCa patients.

## Data Availability

All RNA-sequencing data reported here is available from NCBI-GEO (www.ncbi.nlm.nih.gov/geo) under the following accession numbers: GSE194420, GSE195632, and GSE195624.

## Supplementary Materials

The following supporting information can be downloaded at: https://www.mdpi.com/article/10.3390/cancers14205148/s1, Figure S1: Full annotated Western blot images for LNCaP nuclear and cytoplasmic expression −/+ R1881; Figure S2: Full annotated Western blot images for LNCaP:C4-2 nuclear and cytoplasmic expression −/+ R1881; Figure S3: Full annotated Western blot images for 22Rv1 nuclear and cytoplasmic expression −/+ R1881; Figure S4: Full annotated Western blot images for LNCaP −/+ R1881 METTL3 expression; Figure S5: Full annotated Western blot images for LNCaP:C4-2 −/+ R1881 METTL3 expression; Figure S6: Full annotated Western blot images for 22Rv1 −/+ R1881 METTL3 expression; Figure S7: Full annotated Western blot images for 22Rv1 −/+ STM2457 AR expression; Figure S8: Full annotated Western blot images for LNCaP:C4-2 −/+ siMETTL3; Figure S9: Full annotated Western blot images for 22Rv1 −/+ siMETTL3; Figure S10: METTL3 expression in primary therapy response in PCa patients; Figure S11: siRNA-mediated depletion of METTL3 regulates transcriptional and alternative splicing programmes in PCa cell lines; Table S1: Patient demographics of the prostate TMA cohort; Table S2: Summary of clinical correlations with METTL3 protein expression in the prostate TMA cohort; Table S3: Significantly (fold change ±1.5 and FDR-corrected *p*-value < 0.05) DEGs between Veh and STM2457 treatment in LNCaP:C4-2 and 22Rv1; Table S4: Enriched KEGG pathways with DEGs with METTL3 inhibition using STM2457 in LNCaP:C4-2 and 22Rv1; Table S5: Significant (dPSI ≥ 5% and FDR < 0.05) differential splicing events in Veh vs. STM2457 treatment in LNCaP:C4-2; Table S6: Significant (dPSI ≥ 5% and FDR < 0.05) differential splicing events in Veh vs. STM2457 treatment in 22Rv1; Table S7: Significantly (fold change ±1.5 and FDR-corrected *p*-value < 0.05) DEGs in siSCR vs. siMETTL3 Veh treatment in LNCaP:C4-2 and 22Rv1; Table S8: Enriched KEGG pathways with DEGs with Veh treated siMETTL3 knockdown in LNCaP:C4-2 and 22Rv1; Table S9: Significant (dPSI ≥ 5% and FDR < 0.05) differential splicing events with siSCR vs. siMETTL3 Veh treatment in LNCaP:C4-2; Table S10: Significant (dPSI ≥ 5% and FDR < 0.05) differential splicing events with siSCR vs. siMETTL3 Veh treatment in 22Rv1; Table S11: Significantly (fold change ±1.5 and FDR-corrected *p*-value < 0.05) DEGs with siSCR Veh vs. R1881 and siMETTL3 Veh vs. R1881 in LNCaP:C4-2; Table S12: Enriched KEGG pathways with DEGs with siSCR Veh vs. R1881 and siMETTL3 Veh vs. R1881 in LNCaP:C4-2; Table S13: Significantly (fold change ±1.5 and FDR-corrected *p*-value < 0.05) DEGs with siSCR Veh vs. R1881 and siMETTL3 Veh vs. R1881 in 22Rv1; Table S14: Significantly (fold change ±1.5 and FDR-corrected *p*-value < 0.05) DEGs with siSCR Veh vs. R1881 and siMETTL3 Veh vs. R1881 in 22Rv1; Table S15: Significant (dPSI ≥ 5% and FDR < 0.05) differential splicing events with siSCR Veh vs. R1881 in LNCaP:C4-2; Table S16: Significant (dPSI ≥ 5% and FDR < 0.05) differential splicing events with siMETTL3 Veh vs. R1881 in LNCaP:C4-2; Table S17: Significant (dPSI ≥ 5% and FDR < 0.05) differential splicing events with siSCR Veh vs. R1881 in 22Rv1; Table S18: Significant (dPSI ≥ 5% and FDR < 0.05) differential splicing events with siMETTL3 Veh vs. R1881 in 22Rv1.
