# Supplementary material for: The METTL3 RNA Methyltransferase Regulates Transcriptional Networks in Prostate Cancer
Source: Cancers (Basel). 2022 Oct 20;14(20):5148. doi: 10.3390/cancers14205148 (PMC9600477; doi:10.3390/cancers14205148)
Supplement: Supplementary file 1 [file cancers-14-05148-s001.zip › cancers-1895905-supplementary materials.pdf]

cancers

MDPI

Supplementary Materials

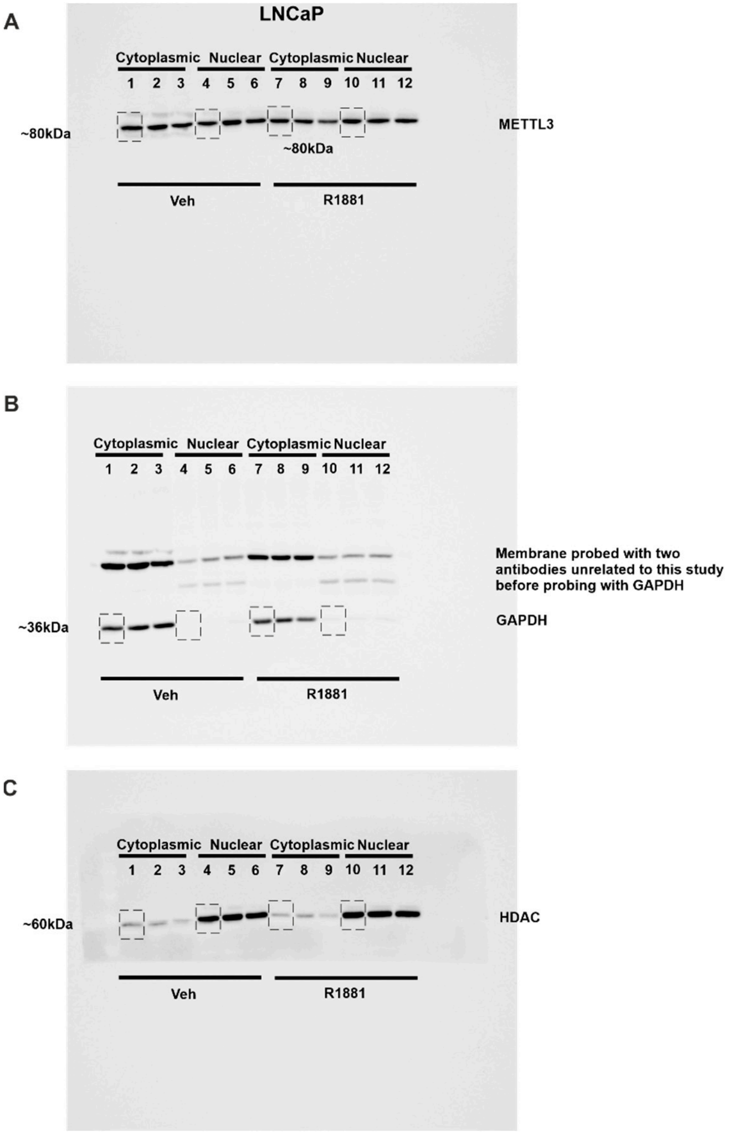

Figure S1. Full annotated western blot images for LNCaP nuclear and cytoplasmic expression -/+ R1881 shown in main Figure 2A. METTL3 (A), GAPDH (B), and HDAC (C) were probed on membranes with samples run from Veh treated cytoplasmic (lanes 1-3) and nuclear fraction (lanes 4-6) and R1881 treated cytoplasmic (lanes 7-9) and nuclear (lanes 10-12) fractions. Lanes indicated by a dashed box (1, 4, 7, and 10) were used as representative bands to display in Figure 2A. Veh = vehicle.

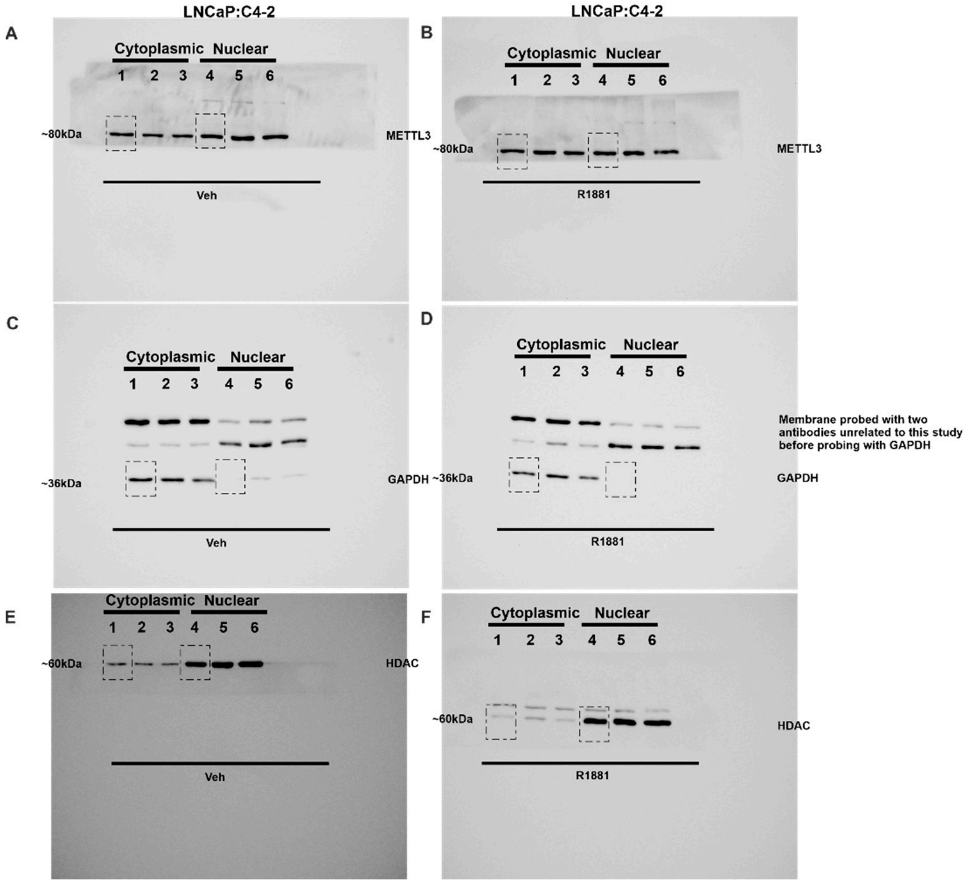

Figure S2. Full annotated western blot images for LNCaP:C4-2 nuclear and cytoplasmic expression -/+ R1881 shown in main Figure 2A. METTL3 (A and B), GAPDH (C and D), and HDAC (E and, F) were probed on membranes with samples run from Veh treated cytoplasmic (A, C, and E lanes 1-3) and nuclear fraction (A, C, and D lanes 4-6) and R1881 treated cytoplasmic (B and D, F lanes 1-3) and nuclear (B, D, and F lanes 4-6) fractions. Lanes indicated by a dashed box (1, 4, 7, and 10) were used as representative bands to display in Figure 2A. Veh = vehicle.

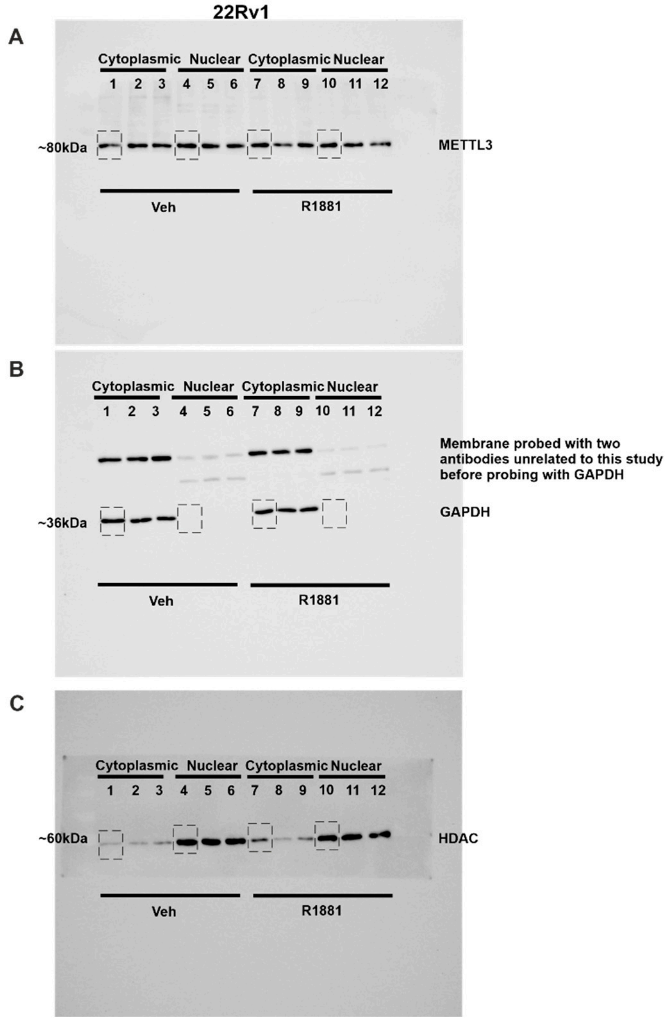

Figure S3. Full annotated western blot images for 22Rv1 nuclear and cytoplasmic expression -/+ R1881 shown in main Figure 2A. METTL3 (A), GAPDH (B), and HDAC (C) were probed on membranes with samples run from Veh treated cytoplasmic (lanes 1-3) and nuclear fraction (lanes 4-6) and R1881 treated cytoplasmic (lanes 7-9) and nuclear (lanes 10-12) fractions. Lanes indicated by a dashed box (1, 4, 7, and 10) were used as representative bands to display in Figure 2A. Veh = vehicle.

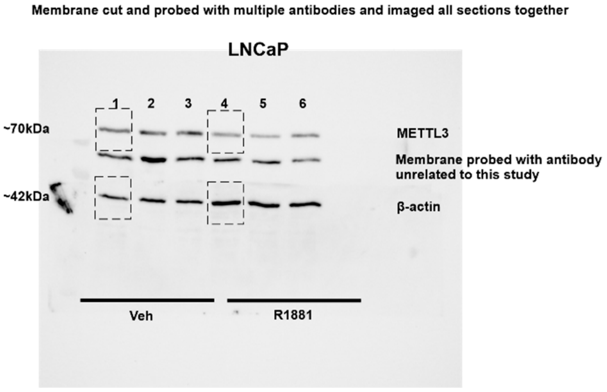

Figure S4. Full annotated western blot images for LNCaP -/+ R1881 METTL3 expression shown in main Figure 2C. The membrane was cut into sections and METTL3 and β-actin were probed on

separate sections of the membrane and imaged together with samples run from Veh treated (lanes 1-3) and R1881 treated (lanes 4-6) LNCaP. Lanes indicated by a dashed box (1 and 4) were used as representative bands to display in Figure 2C. Veh = vehicle.

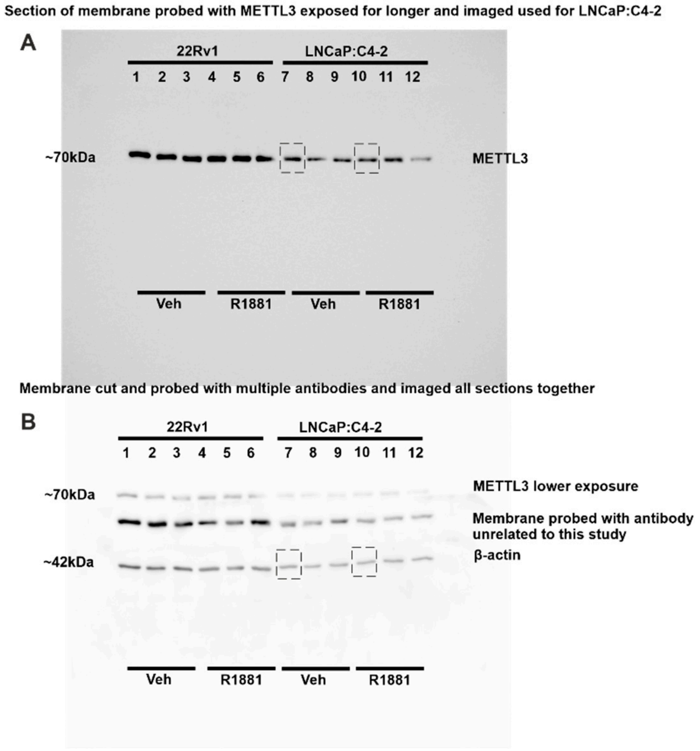

Figure S5. Full annotated western blot images for LNCaP:C4-2 -/+ R1881 METTL3 expression shown in main Figure 2C. The membrane was cut into sections and METTL3 and β-actin were probed on separate sections. (A) Section of membrane probed with METTL3 imaged alone. (B) separate sections of membrane probed with METTL3, β-actin, and antibody unrelated to this study and imaged together. Samples on this blot are 22Rv1 veh (lanes 1-3) and R1881 treated (lanes 4-6) and LNCaP:C4-2 Veh (lanes 7-9) and R1881 treated (lanes 10-12). Lanes indicated by a dashed box (7 and 10) were used as representative bands for LNCaP:C4-2 to display in Figure 2C. Veh = vehicle.

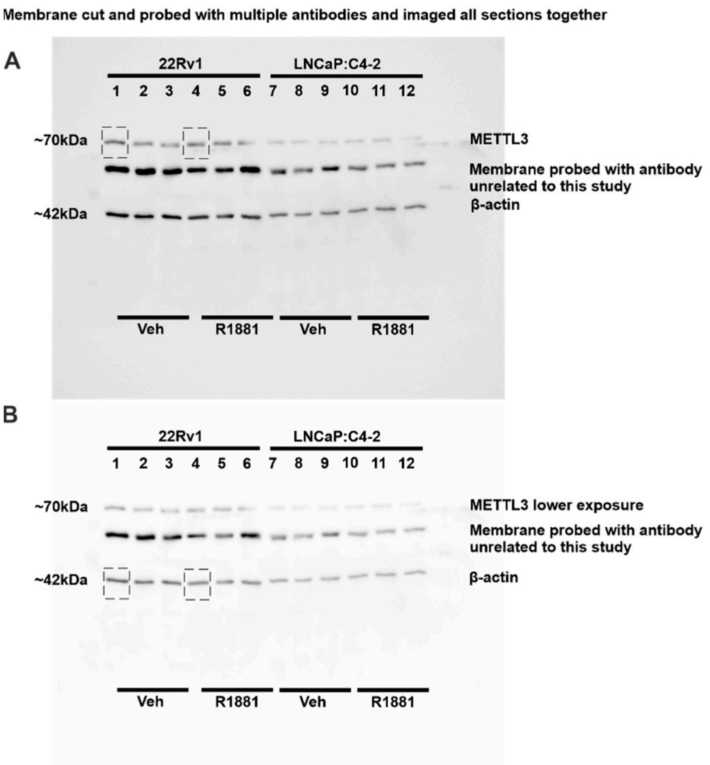

Figure S6. Full annotated western blot images for 22Rv1 -/+ R1881 METTL3 expression shown in main Figure 2C. The membrane was cut into sections and METTL3 and β-actin were probed on separate sections. (A) separate sections of membrane probed METTL3, β-actin, and antibody unrelated to this study and imaged together. (B) separate sections of membrane probed with METTL3, β-actin, and antibody unrelated to this study and imaged together with a lower exposure. Samples on this blot are 22Rv1 Veh (lanes 1-3) and R1881 treated (lanes 4-6) and LNCaP:C4-2 Veh (lanes 7-9) and R1881 treated (lanes 10-12). Lanes indicated by a dashed box (1 and 4) were used as representative bands for 22Rv1 to display in Figure 2C. Veh = vehicle.

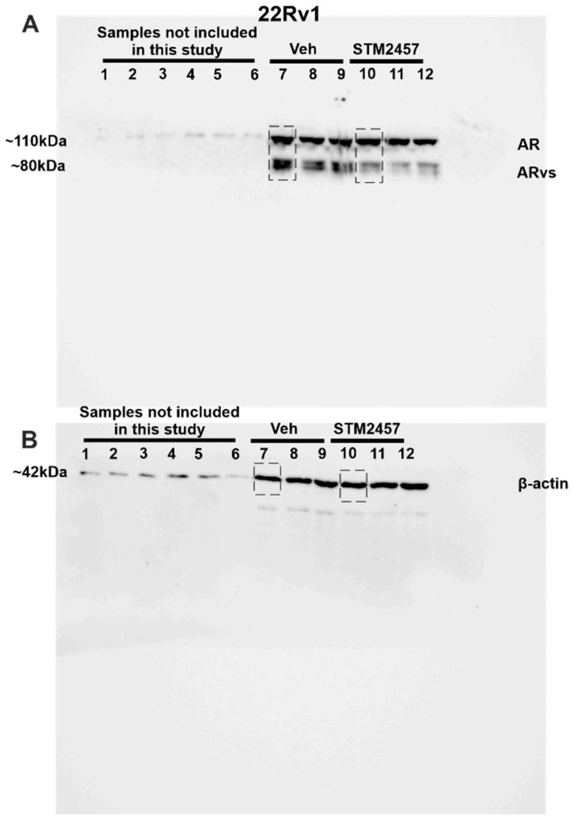

Figure S7. Full annotated western blot images for 22Rv1 -/+ STM2457 AR expression shown in main Figure 3G. The membrane was probed AR (A) and β-actin (B) Samples on this blot are 22Rv1 Veh (lanes 7-9) and STM2457 treated (lanes 10-12). Lanes indicated by a dashed box (7 and 10) were used as representative bands for 22Rv1 to display in Figure 3G. Veh = vehicle.

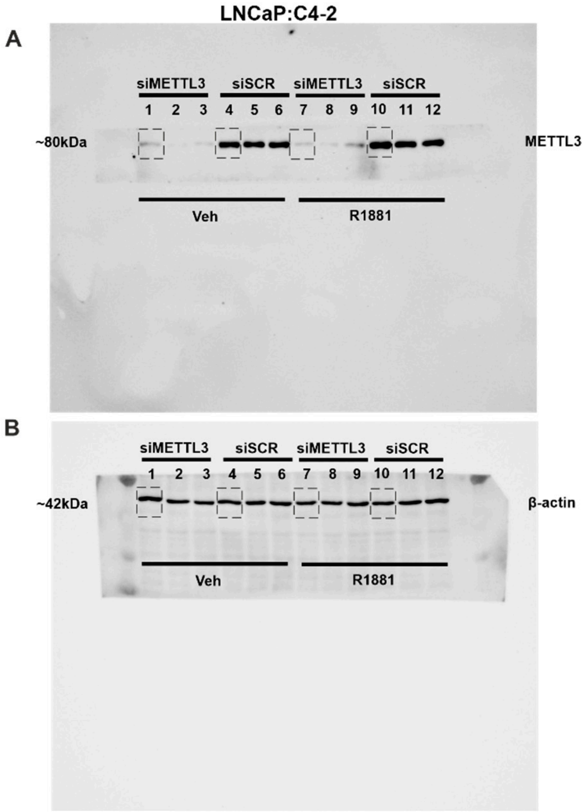

Figure S8. Full annotated western blot images for LNCaP:C4-2 -/+ siMETTL3 shown in main Figure 4A. The membrane was cut into sections and METTL3 (A) and β-actin (B) were probed on separate sections. Samples loaded on this blot are LNCaP:C4-2 Veh treated siMETTL3 (lanes 1-3) and siSCR (lanes 4-6) and R1881 treated siMETTL3 (lanes 7-9) and siSCR (lanes 10-12). Lanes indicated by a dashed box (1, 4, 7, and 10) were used as representative bands to display in Figure 4A. Veh = vehicle.

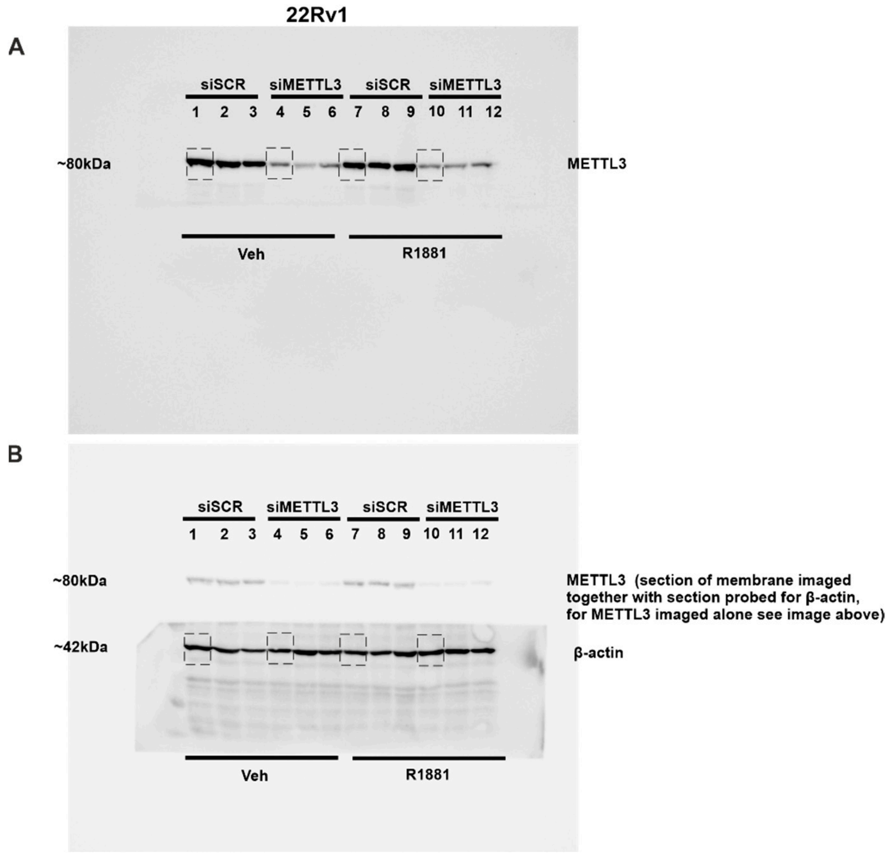

Figure S9. Full annotated western blot images for 22Rv1 -/+ siMETTL3 shown in main Figure 4B. The membrane was cut into sections and METTL3 (A) and β-actin (B) were probed on separate sections. Samples loaded on this blot are LNCaP:C4-2 Veh treated siSCR (lanes 1-3) and siMETTL3 (lanes 4-6) and R1881 treated siSCR (lanes 7-9) and siMETTL3 (lanes 10-12). Lanes indicated by a dashed box (1, 4, 7, and 10) were used as representative bands to display in Figure 4B. Veh = vehicle.

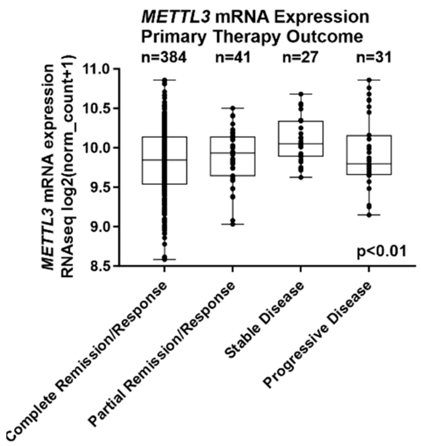

Figure S10. *METTL3* expression in primary therapy response in PCa patients. The TCGA prostate adenocarcinoma was dataset analysed to correlate *METTL3* mRNA primary therapy outcome.

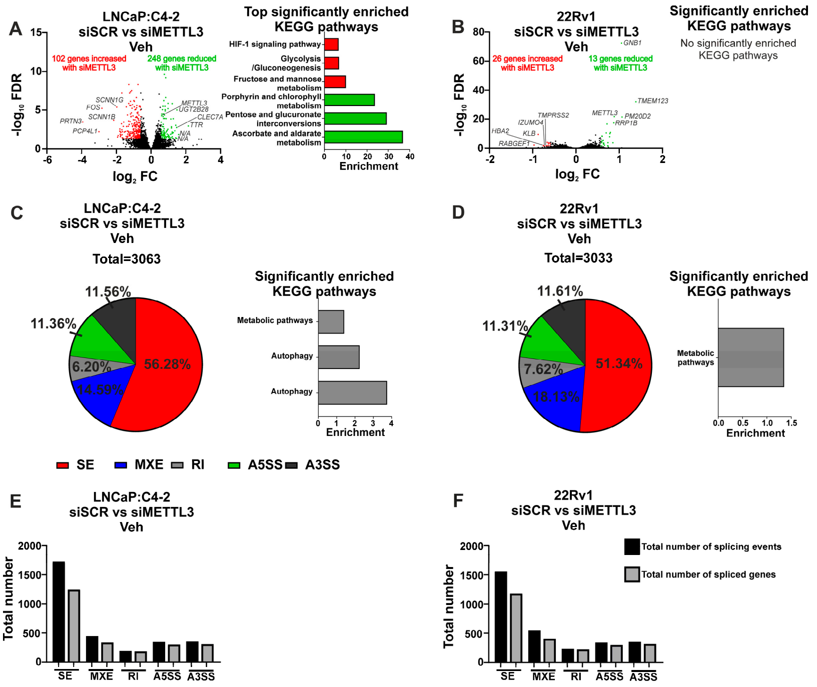

Figure S11. siRNA-mediated depletion of *METTL3* regulates transcriptional and alternative splicing programmes in PCa cell lines. PCa cells treated with siSCR or siMETTL3 under vehicle conditions were analysed by RNA-seq and the DEGs and DSGs determined. The METTL3 regulated differential gene expression and significantly enriched KEGG pathways in LNCaP:C4-2 (A) and 22Rv1 (B) are shown. Genes with significantly higher expression with *METTL3* knockdown are coloured red and the genes significantly lower with *METTL3* knockdown are coloured green. Non-significantly differentially expressed genes are plotted in black. METTL3-regulated differential splicing events and significantly enriched KEGG pathways in siSCR vs. siMETTL3 in LNCaP:C4-2 (C and E) and 22Rv1 (D and F) are shown. Significant gene expression: FC ±1.5 and FDR < 0.05. Significant splicing events: dPSI ≥5% and FDR < 0.05. Veh = vehicle, FC = fold-change, FDR = false discovery rate. SE = skipped exon, MXE = mutually exclusive exon, RI = retained intron, A5SS = alternative 5′ splice site, A3SS = alternative 3′ splice site.
